# Knit-Pix2Pix: An Enhanced Pix2Pix Network for Weft-Knitted Fabric Texture Generation

**DOI:** 10.3390/s26020682

**Published:** 2026-01-20

**Authors:** Xin Ru, Yingjie Huang, Laihu Peng, Yongchao Hou

**Affiliations:** 1 College of Mechanical Engineering, Zhejiang Sci-Tech University, Hangzhou 310018, China; 2023220503046@mails.zstu.edu.cn (Y.H.); laihup@zstu.edu.cn (L.P.); ychao_hou@163.com (Y.H.); 2 Zhejiang Provincial Innovation Center of Advanced Textile Technology, Shaoxing 312030, China; 3 Zhejiang Key Laboratory of Intelligent Manufacturing Equipment for Flexible Functional Materials, Zhejiang Sci-Tech University, Hangzhou 310018, China

**Keywords:** texture mapping, weft-knitted fabrics, generative adversarial networks, fabric simulation

## Abstract

Texture mapping of weft-knitted fabrics plays a crucial role in virtual try-on and digital textile design due to its computational efficiency and real-time performance. However, traditional texture mapping techniques typically adapt pre-generated textures to deformed surfaces through geometric transformations. These methods overlook the complex variations in yarn length, thickness, and loop morphology during stretching, often resulting in visual distortions. To overcome these limitations, we propose Knit-Pix2Pix, a dedicated framework for generating realistic weft-knitted fabric textures directly from knitted unit mesh maps. These maps provide grid-based representations where each cell corresponds to a physical loop region, capturing its deformation state. Knit-Pix2Pix is an integrated architecture that combines a multi-scale feature extraction module, a grid-guided attention mechanism, and a multi-scale discriminator. Together, these components address the multi-scale and deformation-aware requirements of this task. To validate our approach, we constructed a dataset of over 2000 pairs of fabric stretching images and corresponding knitted unit mesh maps, with further testing using spring-mass fabric simulation. Experiments show that, compared with traditional texture mapping methods, SSIM increased by 21.8%, PSNR by 20.9%, and LPIPS decreased by 24.3%. This integrated approach provides a practical solution for meeting the requirements of digital textile design.

## 1. Introduction

Weft-knitted fabrics, owing to their excellent elasticity, breathability, and comfort, are widely used in apparel and home textiles. With the increasing demand for weft-knitted products, there has been a parallel rise in the need for realistic simulation of fabric appearance and physical behavior in fields such as fashion design, film and animation, and game development. This need is particularly evident in achieving accurate representations of elastic deformation and surface textures unique to weft-knitted structures. However, modeling such fabrics presents significant technical challenges. On the one hand, yarn-level models can faithfully capture the microscopic structure of textiles, but their intricate geometries, multi-layered interlacing patterns, and prohibitive computational costs render them impractical for real-time applications. On the other hand, spring-mass models offer higher computational efficiency and can quickly approximate the overall deformation of fabrics, yet the resulting surfaces often lack realistic texture details and visual fidelity, which limits their practical applicability. To resolve this contradiction, researchers have sought approaches that strike a balance between computational efficiency and visual realism. With the rapid advancement of computer graphics, texture mapping methods have been increasingly adopted to enhance the appearance realism of fabric models. For instance, by employing spring-mass or shell models to compute the macroscopic deformation of fabrics while projecting pre-generated textures onto the model surfaces, these methods—such as diffuse mapping, normal mapping, displacement mapping, and specular mapping—have, to some extent, improved the perceptual realism of fabric models. Nevertheless, knitted fabrics are flexible materials with distinctive microstructures and undergo complex geometric transformations of loop configurations during large-scale deformations. For instance, under bidirectional stretching, yarns increase in length while decreasing in thickness, and loops gradually tighten. Traditional texture mapping techniques, which typically rely on geometric transformations of pre-generated textures to fit deformed surfaces, neglect these structural variations. The mismatch between the underlying physical mechanisms and the visual representations becomes particularly pronounced under large deformations, thereby severely compromising the visual realism of knitted fabric models.

In recent years, some researchers have adopted a pre-computed texture mapping strategy [1]. This approach pre-calculates and stores the fabric appearance under different stretching and compression states, establishing a mapping between deformation parameters and texture variations. At runtime, textures are retrieved through real-time computation. However, such methods are computationally expensive. With the rapid progress of deep learning, Generative Adversarial Networks (GANs) have achieved wide adoption in computer vision due to their outstanding image generation capabilities. From the original GAN [2] designed for unconditional image generation to derivative models such as Cycle GAN [3] for image style transfer, GAN variants have opened new technical pathways for solving image generation tasks. Among them, the Pix2Pix model [4], based on conditional GANs, can establish explicit input–output mappings. Ahme et al. [5] successfully applied this model to convert user sketches into stylized color patterns. This property also makes Pix2Pix well-suited for our task of generating fabric textures from knitted unit mesh maps.

However, the traditional Pix2Pix model exhibits clear limitations when applied to knitted-fabric textures. A careful analysis of the structural characteristics of knitted textures and their corresponding knitted unit mesh maps reveal a pronounced multi-scale hierarchy: at the local scale, mesh lines and intersection nodes define individual loop geometries; at the meso scale, the regular arrangement of neighboring mesh cells gives rise to periodic knitting patterns; and at the macro scale, large-range mesh deformations encode the fabric’s global geometric variations. These multi-scale features correspond to different levels of information that must be synthesized—ranging from loop-level fine detail, through repeating knit motifs, to the overall deformation state.

The standard Pix2Pix architecture, which relies on a single convolutional pathway with a fixed receptive field, is not well suited to model such hierarchical information. A small fixed receptive field is limited to local mesh strokes, failing to capture inter-cell repetition and long-range deformation patterns. In contrast, a large, fixed receptive field can recover global geometry but often blurs fine local features and loses loop-level detail. Processing all scales within the same feature space further induces cross-scale interference and diminishes the model’s representational capacity. As a result, these architectural constraints manifest as structural discontinuities and indistinct local details in the generated knitted textures.

To address this issue, we propose Knit-Pix2Pix, an integrated framework for weft-knitted fabric texture generation, based on a Pix2Pix architecture. First, to overcome the fixed receptive field of the original Pix2Pix, which often causes detail loss and texture artifacts, the framework employs multi-scale generator and discriminator networks. In the generator, convolutional blocks with different receptive fields process multi-scale features in parallel, and the discriminator extracts the discriminative features of multiple levels via input branches with different scaling ratios. Furthermore, since the original network lacks the ability to learn the complex mapping between mesh deformations and texture variations, the framework incorporates a grid-guided attention mechanism, where mesh deformation information is used as prior knowledge to guide the model in capturing this mapping more precisely. This integrated design allows the framework to effectively address the practical challenges in knitted fabric texture synthesis.

The remainder of this paper is organized as follows: Section 2 reviews related work; Section 3 details the proposed method; Section 4 describes dataset preparation; Section 5 presents and analyzes the experimental results; Section 6 and Section 7 discuss the limitations and advantages of our method and concludes the full paper and future research directions.

## 2. Related Work

### 2.1. Yarn-Level Simulation

Yarn-level simulation methods achieve high-fidelity fabric dynamics by accurately modeling yarn physics. Kaldor et al. [6] were the first to systematically propose a yarn-level model for fabric simulation. They represented each yarn as an inextensible but deformable B-spline tube. Yarn-yarn friction was approximated using rigid-body velocity filters, and interactions were modeled through hard constraint forces. This approach successfully reproduced the nonlinear characteristics and mechanical behavior of fabrics. It captured microscopic details with high accuracy, but the computational cost was immense, making it unsuitable for real-time interactive applications. To improve efficiency, Cirio et al. [7,8,9] assumed permanent but slidable contact between yarns. By completely eliminating collision detection, their method reduced computational cost and achieved an order-of-magnitude speedup over earlier approaches, while still preserving key fabric properties. However, this permanent-contact assumption may fail to capture realistic behavior in some fabric types, especially in loosely knitted structures. Ru et al. [10] used Catmull–Rom splines to fit yarn centerlines and employed motion vectors to represent four fundamental stitches (plain, tuck, float, and transfer). They then solved the Euler–Lagrange equations to compute yarn control points. This work effectively addressed yarn-level modeling challenges for patterned weft-knitted fabrics. Yuksel et al. [11] represented large-scale knitted surfaces with polygonal meshes and combined them with yarn-level models to simulate the deformation of complex knitting patterns, enabling realistic 3D knitted garment simulation. Overall, yarn-level simulation captures fabric physics and microstructural details well but suffers from high computational complexity and low efficiency, making it difficult to balance performance with visual realism.

### 2.2. Texture Mapping

Texture mapping techniques map appearance information such as texture and color onto planar or loop geometry models to reproduce basic fabric appearance. These methods are computationally inexpensive and relatively simple, making them well-suited for applications requiring fast visualization. Lu Z et al. [12] proposed a fast simulation method combining yarn textures with loop geometry. They divided each loop into six regions and built a 2D mapping from straight yarn textures to curved loops. This model quickly generated realistic plain and rib fabrics. Jiang et al. [13] proposed a plain-knit deformation method based on an interlacing-point model. They applied texture mapping along loop centerlines, used four-point interpolation to fill missing textures, and processed brightness variations, resulting in more realistic deformed loops. Wu Y et al. [14] tackled the unpredictable effects of mélange and cloud yarn fabrics by mapping processed real yarn images onto loop geometry. By computing offsets during loop stitching, they resolved misalignment issues and simulated realistic cloud-yarn knits, enabling appearance prediction for specialty yarn fabrics. Due to the difficulty of predicting the cloth effect of color spun yarn and cloudy yarn, Wu, Y et al. [15] processed the real yarn image and mapped it onto the coil geometry model, solved the problem of splicing misalignment by calculating the offset when splicing the coils and simulated cloudy yarn knitted fabrics with a high level of realism, which effectively achieves the purpose of predicting the appearance of knitted fabrics made of special yarns. Sperl et al. [16] realized dynamic simulation of weft-knitted fabrics at the yarn level using numerical homogenization. They built a potential energy density model from large-scale yarn-level simulations and computed fabric mechanics in a thin-shell simulator. This approach captured high deformability and anisotropy. but the authors still used prefabricated texture maps to simulate the appearance of the fabric model. As a result, loop structures did not deform physically with fabrics, creating a clear mismatch between visual and physical behavior under large deformations. The above mapping methods mainly focus on the restoration of the macroscopic visual effect of the fabric, which can present the basic appearance characteristics of the fabric, but lacks the accurate expression of the detailed changes in the texture of the fabric. The method proposed in this paper effectively solves the problem, and can generate corresponding textures according to different macroscopic deformation states of fabrics.

### 2.3. Generative Adversarial Network

In recent years, GAN-based image generation has been extensively studied. GANs achieve high-quality image synthesis through adversarial training between a generator and a discriminator. In the research direction of GAN, some typical variants have been proposed, including WGAN-GP [17], StyleGAN3 [18], Diffusion-GAN [19], Vit-VQGAN [20]. In addition, many conditional GAN (CGAN) [21] models have been introduced to control image generation under specific conditions. Conditional inputs include class labels [22], attributes [23], segmentation maps [24,25,26], texture patches [27,28], image examples [29,30,31], and text descriptions [32]. These conditions provide additional guidance to improve generation performance.

Image-to-image translation is a GAN-based research direction that learns mappings between source and target domains. These studies fall into two categories. The first is supervised, requiring paired data. Pix2Pix was the first conditional GAN model for supervised image-to-image translation, handling tasks such as day-to-night, labels-to-street, and aerial-to-map. Building on it, Pix2PixHD [33] was later proposed for high-resolution generation. The second is unsupervised, which does not require paired data. These methods rely on cycle consistency for cross-domain translation. Representative models include Cycle GAN, GP-UNIT [34], MUNIT [35], Star GAN [36], and SPA-GAN [37].

Inspired by the successful application of image-to-image translation techniques in texture synthesis, this study introduces such a framework into the task of weft-knitted fabric texture generation. The problem, however, presents unique challenges: the periodic patterns inherent in weft-knitted structures, the intricate geometric interlacing of yarns, and the diverse texture variations under different deformation states collectively impose higher demands on generative models. To address these issues, we propose Knit-Pix2Pix, an enhanced Pix2Pix-based approach specifically tailored for knitted texture generation, which enables accurate synthesis of realistic fabric appearances directly from knitted unit mesh maps.

## 3. Method

### 3.1. Network Architecture

**Figure 1:**
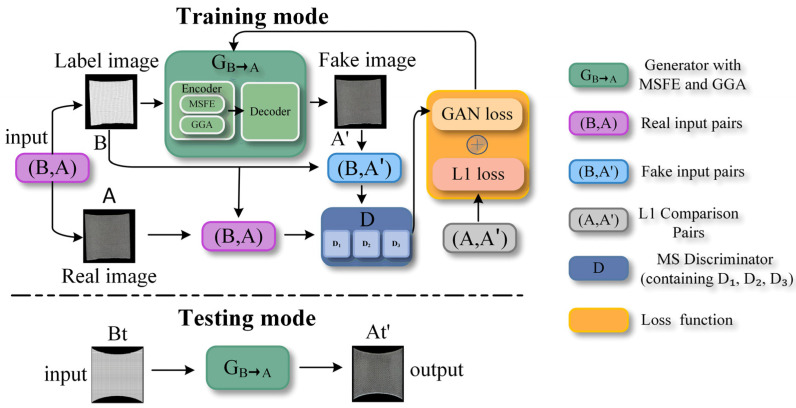
Workflow of the network.

The proposed weft-knitted texture generation framework, as illustrated in Figure 1, adopts a mesh-to-texture (B2A) conversion mode. In this mode, the knitted unit mesh maps (B) are transformed into realistic fabric texture images (A), aiming to establish a direct relationship between the geometric deformation of the fabric structure and its resulting visual appearance. Specifically, the knitted unit mesh B serves as a structural guide, in which each cell corresponds to a physical loop region and its deformation state, while the output A represents the photorealistic texture that should appear under such deformation. By learning this mapping, the model bypasses the limitations of traditional texture warping. To effectively achieve this complex mapping, the generator G is enhanced with two core components: a Multi-Scale Feature Extraction (MSFE) module and a Grid-Guided Attention (GGA) mechanism. The MSFE module is designed to overcome the limitation of a single receptive field in the standard U-Net, which is ill-suited for capturing the hierarchical features of knitted structures—ranging from local loop geometry to global deformation patterns. Its parallel architecture with different dilation rates allows for simultaneous and non-interfering perception of micro, meso, and macro features, bringing the benefit of comprehensive geometric context understanding without the blurring or loss of detail associated with a single large kernel. The GGA mechanism is introduced to explicitly incorporate the structural prior from the knitted unit mesh map. Unlike self-attention that relies solely on feature statistics, GGA uses the knitted unit mesh deformation as direct guidance to spatially recalibrate feature responses. This design enables the generator to focus precisely on regions where deformation most significantly affects texture appearance, thereby enhancing the accuracy of texture synthesis in critical areas. The multi-scale discriminator D employs three sub-discriminators operating at different resolutions to comprehensively assess the realism of the generated textures.

During training, a knitted unit mesh map B is first fed into generator G. The generator processes the mesh map through the Multi-Scale Feature Extraction (MSFE) module, which captures hierarchical geometric patterns from local structures to global deformation states via parallel dilated convolutions with different receptive fields. The extracted multi-scale features are then refined by the Grid-Guided Attention (GGA) mechanism, which leverages the mesh’s structural information to adaptively emphasize deformation-critical regions. Through this process, the generator produces a synthetic texture A′. The generated image A′ and the ground-truth texture A are then jointly passed into the multi-scale discriminator D for authenticity evaluation across multiple scales. Network parameters are iteratively updated by optimizing the adversarial loss, enabling the model to implicitly learn the complex mapping between geometric deformation and visual appearance, including phenomena such as loop morphology changes during stretching. At inference, any knitted unit mesh map can be used as input to generate the corresponding texture.

### 3.2. Improved Generator Design

**Figure 2:**
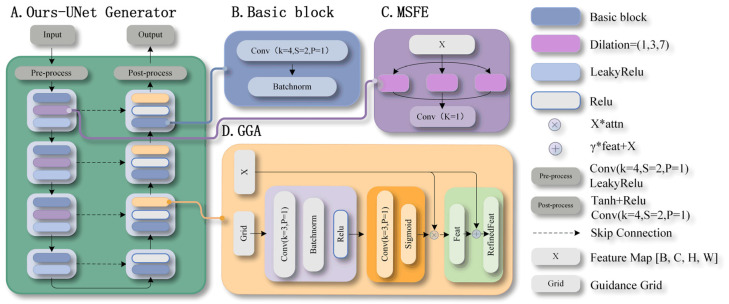
Improved Generator Architecture.

We design an enhanced UNet generator architecture, as shown in Figure 2. Two new modules are introduced on top of the standard UNet: the Multi-Scale Feature Extraction (MSFE) module (Figure 2C) and the Grid-Guided Attention (GGA) mechanism (Figure 2D). The MSFE enhances the network’s perception of multi-scale mesh features via parallel dilated convolutions with different rates. GGA leverages prior information from the knitted unit mesh map to adaptively adjust feature responses, enabling the generator to focus more precisely on texture-critical regions. The generator uses UNet as the backbone, with MSFE and GGA integrated at specific layers. Their design and role in texture synthesis are detailed below.

### 3.3. Multi-Scale Feature Extraction

We design the MSFE module to establish multi-scale receptive fields, allowing the network to process geometric features in parallel. Specifically:

Dilated convolutions expand receptive fields by inserting zeros between kernel elements. With dilation rate r, a 3 × 3 kernel covers an effective receptive field of (3 − 1) × r + 1. Thus r = 1,3,7 correspond to perceptive ranges of 3 × 3,7 × 7,15 × 15 pixels, respectively, This specific sequence of rates is designed to grow by a similar multiplicative factor at each step, which ensures a cohesive and progressive expansion of the receptive field, enabling the network to hierarchically model the fabric structure: from capturing the yarn trajectory and local curvature to the geometry of individual loops, and finally to the inter-loop patterns and structural arrangements within a cell block. Parallel multi-branch processing avoids the trade-off of a single receptive field, while independent branches eliminate cross-scale interference. A feature fusion layer then integrates multi-scale information collaboratively, overcoming the limitations of the original model. Below are the specific workflows.

Given an input feature $F_{in}\in R^{H\times W\times C}$, the MSFE module constructs three parallel processing branches with different expansion rates to capture multi-scale spatial context information. The processing flow is defined as follows:

(1) $$F_{i}^{\left(r\right)}={\mathrm{DilConv}}_{r}\left({\mathrm{Conv}}_{3\times 3}\left(F_{in}\right)\right)\;r\in \{1,3,7\},i\in \{1,2,3\}$$

where ${\mathrm{DilConv}}_{r}$ denotes a dilated convolution with rate r. This design enables the module to perceive structural information at multiple scales, providing layered feature representations for texture synthesis.

Finally, outputs from all branches are fused into a unified representation via a 1 × 1 convolution. This layer adaptively learns weights for different scales, ensuring effective collaboration among structural features:

(2) $$F_{out}=\mathrm{Fusion}\left(F_{1}^{\left(1\right)},F_{2}^{\left(3\right)},F_{3}^{\left(7\right)}\right)={\mathrm{Conv}}_{1\times 1}\left(\mathrm{Concat}\left(F_{1}^{\left(1\right)},F_{2}^{\left(3\right)},F_{3}^{\left(7\right)}\right)\right)$$

where Concat (⋅) denotes concatenation along the channel dimension.

### 3.4. Grid-Guided Attention (GGA)

To strengthen the model’s ability to learn the mapping between fabric deformation and texture variation, we introduce the GGA module and integrate it into the middle layers of the decoder. Unlike traditional attention mechanisms that rely on information about the feature map itself, GGA learns spatial structural patterns from the knitted unit mesh map, enabling the generator to attend more precisely to texture-critical regions. GGA takes two inputs: the feature map to be enhanced $X\in R^{H\times W\times C}\;$ and a mesh mask $\mathrm{Grid}\in R^{H\times W\times 1}$. The mesh mask serves as guidance for accurate spatial attention computation. The overall formulation of GGA is:

(3) $$GGA\left(X,\mathrm{Grid}\right)=\gamma \cdot \left(X⊙\sigma \left({\mathrm{Conv}}_{8\to 1}\left(ReLU\left(BN\left({\mathrm{Conv}}_{1\to 8}\left(\mathrm{Grid}\right)\right)\right)\right)\right)\right)+X$$

Firstly, the grid encodes $f_{enc}$ Feature extraction for knitted unit mesh map:

(4) $${\mathrm{Grid}}_{feat}=f_{enc}\left(\mathrm{Grid}\right)=\mathrm{ReLU}\left(\mathrm{BN}\left({\mathrm{Conv}}_{1\to 8}\left(\mathrm{Grid}\right)\right)\right)$$

where ${\mathrm{Conv}}_{1\to 8}$ denotes a 3 × 3 convolution expanding channels from 1 to 8, BN is batch normalization, and ReLU is the activation. The encoder uses bias-free convolutions and BN to stabilize training while efficiently capturing structural patterns in the mesh mask. Providing guidance information for subsequent attention calculations.

Next, the encoded mesh features ${\mathrm{Grid}}_{feat}\in R^{H\times W\times 8}$ are transformed into a spatial attention map:

(5) $$A=\sigma \left({\mathrm{Conv}}_{8\to 1}\left({\mathrm{Grid}}_{feat}\right)\right)$$

where $A\in R^{H\times W\times 1}$ is the generated spatial attention map, σ is the Sigmoid function, constraining attention to [0, 1], 0 means completely suppressing the feature at that position, while 1 means fully retaining it. ${\mathrm{Conv}}_{8\to 1}$ denotes a 3 × 3 convolution that reduces the channel dimension from 8 to 1 to produce the single-channel attention map.

This way, the model can learn the importance of different regions in the mesh structure, enabling selective feature enhancement in the spatial dimension. Then, the spatial attention map is element-wise multiplied with the input feature map to enhance the features. When the spatial dimensions of the attention map and feature map do not match, bilinear interpolation is used to resize the attention map:

(6) $$X_{refined}=X⊙\mathrm{Resize}\left(A\right)$$

where ⊙ denotes element-wise multiplication (Hadamard product), and Resize (⋅) is the bilinear interpolation operation.

Finally, to ensure stability and facilitate progressive learning, GGA uses a residual gate with a learnable strength:

(7) $$X_{out}=\gamma \cdot X_{refined}+X$$

where γ is a learnable strength parameter, initialized to 0. During training, γ is updated automatically via backpropagation as a network parameter, and its gradient is calculated as:

(8) $$\frac{\partial L}{\partial \gamma }=\frac{\partial L}{\partial X_{out}}\cdot X_{refined}$$

This design helps the model remain stable during the early stages of training, gradually introducing the effect of the attention mechanism as γ increases. This progressive learning strategy effectively avoids instability that might arise from attention mechanisms in the early stages while ensuring the complete transmission of original feature information.

### 3.5. Multi-Scale Discriminator (MSD)

**Figure 3:**
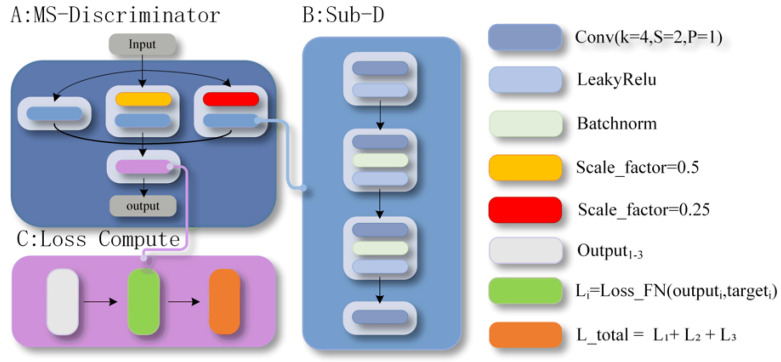
Improved Discriminator Architecture.

To provide comprehensive supervision across different spatial levels, we adopt a Multi-Scale Discriminator (MSD) architecture inspired by Pix2PixHD, as shown in Figure 3. The MSD consists of three parallel sub-discriminators operating at resolution scales of 1.0, 0.5, and 0.25, respectively. This design enables simultaneous evaluation of fine texture details, meso-scale pattern continuity, and global deformation plausibility. The full-resolution discriminator focuses on local texture fidelity, such as yarn micro-structures and edge sharpness. The intermediate-scale discriminator emphasizes the consistency of periodic knitting patterns and transitions between neighboring regions, while the lowest-resolution discriminator assesses the perceptual realism of global deformation trends. Each sub-discriminator follows a standard PatchGAN architecture.

Given an input image $I\in R^{H\times W\times C}$, the multi-scale inputs are defined as:

(9) I1=II2=Downsample(I,0.5)I3=Downsample(I,0.25)

The Least Squares GAN (LSGAN) loss is employed for stable adversarial training. The total loss for the multi-scale discriminator is defined as:

(10) $$L_{MSD}=\sum_{i=1}^{3}L_{GAN}\left(D_{i}\left(I_{i}\right)\right)$$

The weight of $L_{GAN}$ is set to 1. Finally, the complete training objective integrates the multi-scale discriminator loss with L1 reconstruction:

(11) $$L_{-}total=L_{MSD}+\lambda \cdot L\_L1=L_{MSD}+\lambda \cdot E_{x,y}\left[|y-G\left(x\right){|}_{1}\right]$$

where λ = 100 following the Pix2Pix framework and the weight of $L_{MSD}\;$ is set to 1.

Compared to traditional single-discriminator architectures, the MSD’s multi-scale strategy provides more comprehensive supervision for the generator, forcing it to optimize output quality across different spatial levels. Moreover, parallel discriminators at different scales avoid potential blind spots in single-scale evaluation, improving the accuracy and robustness of quality assessment for generated samples. Finally, the hierarchical loss weighting design ensures more stable training and prevents over-optimization of specific scale features that could lead to degradation of other scale features.

## 4. Dataset

### 4.1. Data Collection

#### 4.1.1. Training Set

The training dataset for this study was built based on a custom bi-axial stretching experimental platform. This platform integrates a high-precision load cell, a bi-axial stretching fixture system, a high-resolution imaging system, and a uniform lighting system, enabling real-time image acquisition of fabrics under controlled stretching conditions. The selected weft-knitted fabric samples, all composed of polyester fibers, were of four sizes: 15 cm × 15 cm, 25 cm × 25 cm, 30 cm × 30 cm, and 40 cm × 40 cm. The stretching rate was set at 1 cm/s, and the maximum strain was limited to 30% to ensure reversibility of the test and avoid plastic deformation of the fabric.

The experimental procedure follows a standardized protocol: First, the fabric sample is fixed at both ends in the bi-axial stretching fixture, ensuring that the fabric surface is flat and tension is evenly distributed in all directions. Then, a synchronized bi-axial stretching experiment is conducted, from the initial state to a 30% maximum strain. During the entire stretching process, a high-resolution camera continuously captures images at a frequency of 120 frames per second, recording the fabric’s transition from the initial to the maximum deformation state.

To ensure consistency of data quality, all experiments were conducted in a temperature and humidity-controlled environment. Fabric was pre-conditioned before stretching to eliminate initial stress, and real-time monitoring of tensile data ensured the stability of the stretching process. Following this standardized experimental process, more than 2000 high-quality fabric deformation images from four size groups were collected.

Following common practice in deep learning-based image generation tasks and computer vision fields, the dataset was randomly split into training, validation, and test sets. The standard ratio of 70%/15%/15% was adopted, resulting in 1470 pairs for training, 315 for validation, and 315 for testing.

#### 4.1.2. Test Set

To further evaluate the model’s performance, this study employs a spring-mass fabric model to generate the test dataset. The spring-mass model, as a classical physical simulation approach, effectively describes fabric deformation behavior. Moreover, the mesh representation of the spring-mass model exhibits structural consistency with the knitted unit mesh maps, enabling the direct utilization of simulation results as inputs for the deep learning model while providing an ideal data format for subsequent practical applications. Through systematic adjustment of mesh resolution, material parameters, and stretching conditions, simulation mesh sequences covering various deformation states are generated. The final test dataset comprises 315 simulated mesh states, each stored in the standard format of knitted unit mesh maps.

#### 4.1.3. Traditional Texture Mapping Method

To replicate and visualize the fundamental problem in weft-knitted fabric texture mapping. The traditional texture mapping baseline employs a four-point perspective transformation approach to adapt a reference texture onto deformed mesh regions. The method begins by obtaining a reference texture image T_ref from the fabric in its undeformed state, which serves as the source template for subsequent mapping operations. To establish correspondence between the reference and target geometries, four corner points are extracted from both domains. For the reference texture, corner points C_ref = {p_1_, p_2_, p_3_, p_4_} are detected through binary thresholding followed by contour extraction and polygonal approximation. Similarly, the corner points C_tgt are identified from the knitted unit mesh maps using color space segmentation and morphological operations. The detected corners are sorted in a consistent spatial order (top-left, top-right, bottom-right, bottom-left) to ensure proper correspondence between the two coordinate systems. Given these correspondences, a 3 × 3 homography matrix H is computed to establish the projective mapping relationship. The homography satisfies the constraint:

(12) $$\lambda_{i}p_{i}^{\prime }=Hp_{i},\;\;\;\;\;\;i=1,2,3,4$$

where pᵢ ∈ C_ref and p′ᵢ ∈ C_tgt represent corresponding corner points in homogeneous coordinates, and λᵢ are scalar factors. The matrix H is obtained through direct linear transformation by solving the resulting overdetermined system using singular value decomposition.

With the homography determined, the reference texture is warped to match the target geometry through projective transformation:

(13) $$T_{\mathrm{warped}}\left(x^{\prime },y^{\prime }\right)=T_{\mathrm{ref}}\left(H^{-1}{\left[x^{\prime },y^{\prime },1\right]}^{T}\right)$$

where (x′,y′) denotes pixel coordinates in the target region. Bilinear interpolation is employed to compute pixel intensities at non-integer coordinates during the warping process.

Finally, the warped texture is composited onto the target image using a binary mask M derived from the mesh region:

(14) $$I_{\mathrm{output}}\left(x,y\right)=I_{\mathrm{target}}\left(x,y\right)\left(1-M\left(x,y\right)\right)+T_{\mathrm{warped}}\left(x,y\right)M\left(x,y\right)$$

where M (x, y) ∈ {0,1} defines the spatial extent of the target mesh region. The implementation employs a polygonal approximation tolerance of 2% of the contour perimeter for corner detection, and bilinear interpolation for sub-pixel texture sampling.

### 4.2. Data Processing

**Figure 4:**
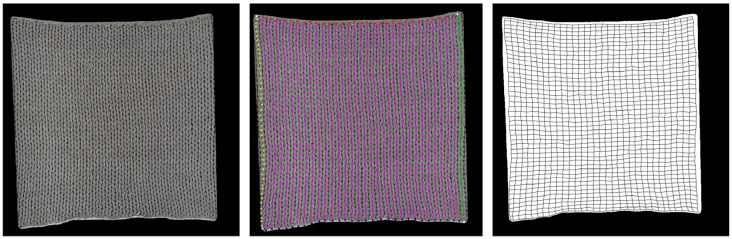
Knitted Unit Mesh Mapping Generation Process.

The initially collected fabric stretching images need to be processed using various image processing techniques to convert them into a standardized format suitable for Pix2Pix model training. First, LabelMe software (version 5.4.1) was used to perform precise contour extraction on each fabric image, generating a binary mask image to clearly define the boundaries of the fabric’s valid region. Next, a gradient-based coil recognition algorithm was employed to automatically locate the center coordinates of each knitted loop in the original image, and these feature points were mapped onto the corresponding mask image. To accurately describe the fabric’s geometric structural features, cubic spline interpolation was used to fit curves through the loop feature points, generating a knitted unit mesh that reflects the fabric’s microstructure. as shown in Figure 4.

## 5. Result

### 5.1. Experimental Data Set and Parameter Environment

The hardware configuration for model training includes an Intel Xeon(R) Platinum 8457C processor and an NVIDIA L20 dual-GPU system. The experimental development environment uses Python 3.7, PyTorch 1.11.0, and CUDA 11.3. All models, including the proposed Knit-Pix2Pix and the baseline models (Pix2Pix, Pix2PixHD, SPADE), were trained from scratch using the same dataset split. In our model, the training configuration was meticulously set as follows: we employed the Adam optimizer (β1 = 0.5, β2 = 0.999, ε = 1 × 10^−8^) with an initial learning rate of 0.0002, which was held constant for the first 100 epochs and not decayed thereafter. The training process was conducted for a total of 200 epochs with a batch size of 2. To quantitatively compare the model complexity and computational efficiency, we present the parameter counts (Params), training time, and inference time for all compared methods in Table 1.

PSNR (Peak Signal-to-Noise Ratio), SSIM (Structural Similarity Index), and LPIPS (Learned Perceptual Image Patch Similarity) were used as objective evaluation metrics. PSNR mainly reflects the pixel-level differences between the reconstructed and original images; the higher the value, the better the image quality. SSIM measures image similarity in terms of brightness, contrast, and structure; a higher SSIM value indicates better image quality. LPIPS, based on feature space distance extracted by deep learning models, reflects human visual perception of image quality, with smaller values indicating higher perceptual quality. These three metrics evaluate image quality from different dimensions, providing complementary perspectives on quality assessment.

### 5.2. Spring-Mass Model Mechanical Consistency Verification

To verify that our spring-mass model exhibits high mechanical consistency with real fabrics, we conducted a comparative validation experiment under equivalent conditions. We selected 5 real fabric samples and subjected them to the same bidirectional tensile forces (ranging from 1N to 10N at 2N intervals) using our biaxial stretching platform. Simultaneously, the spring-mass simulation was configured with identical force magnitudes, loading directions, and boundary conditions to generate corresponding deformed knitted unit mesh maps.

**Figure 5:**
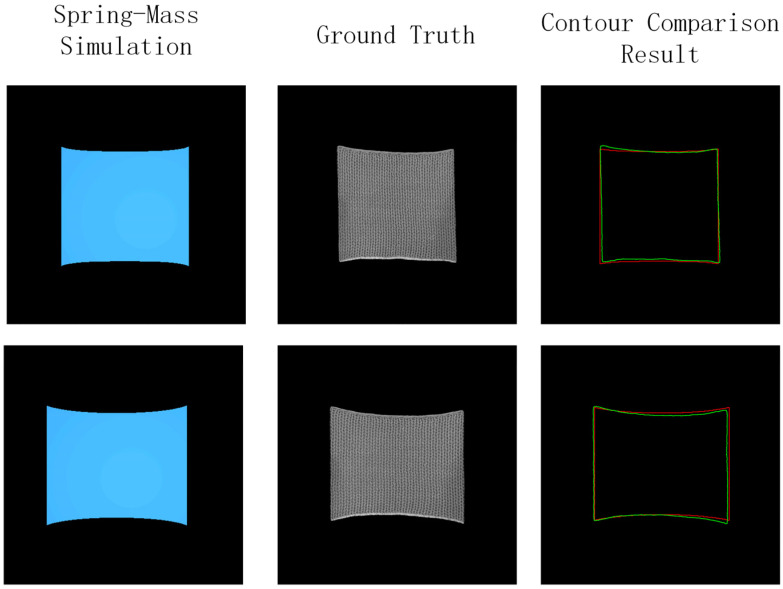
Comparison results between Spring-Mass Model and Ground truth.

Figure 5 presents the contour comparison between real fabric deformations (green) and spring-mass simulated meshes (red) under identical loading conditions. Further quantitative analysis reveals high consistency between the two, with Intersection over Union (IoU) reaching 96.3% ± 1.2%, Dice coefficient of 98.1% ± 0.7%, and overlap ratio of 97.5% ± 1.1%. These consistently high similarity scores (>96%) confirm that our designed spring-mass model can accurately reproduce the mechanical deformation behavior of real weft-knitted fabrics.

### 5.3. Ablation Experiment

To validate the effectiveness of the MSFE module, GGA module, and multi-scale discriminator, we conducted a series of ablation experiments. These experiments were based on the Pix2Pix architecture (denoted as Base) on the same dataset. The configurations of all model variants are detailed in Table 2, Each row corresponds to a distinct model variant, assigned a unique ID from M1 to M5. In the table, a checkmark (✓) denotes the inclusion of a specific module, whereas a cross (✗) indicates its exclusion. The experimental results are shown in Table 2. Experimental results show that the average PSNR and SSIM of the proposed Knit-Pix2Pix framework reach 20.8 and 0.78, respectively, showing a 26.1% and 32.2% improvement over the baseline model, while the LPIPS value decreases to 0.143, improving by 28.5%. These results demonstrate that the proposed network can effectively improve texture image generation and mapping accuracy, and that each module contributes to enhancing network performance. Specifically, the MSFE module expands the network’s receptive field, enhancing its ability to capture both local and global image details. The introduction of the GGA module effectively utilizes prior information from fabric textures, improving the accuracy of texture generation. The multi-scale discriminator enhances the ability to discriminate image details and overall realism, effectively ensuring the visual authenticity and structural consistency of the generated textures. Although the proposed modules increase the model’s parameter count and computational complexity to some extent, they significantly improve key evaluation metrics such as PSNR, SSIM, and LPIPS compared to the baseline Pix2Pix model, proving that this additional computational overhead is both reasonable and necessary.

### 5.4. Comparative Results

To comprehensively evaluate the performance and generalization of the proposed method, we compare the Knit-Pix2Pix model with several baseline methods on two distinct test sets: a held-out Real-Image Test Set and a Spring-Mass Simulation Test Set. This dual evaluation strategy allows us to assess the model’s fidelity to real fabric appearances as well as its capability to handle simulated deformation states.

**Figure 6:**
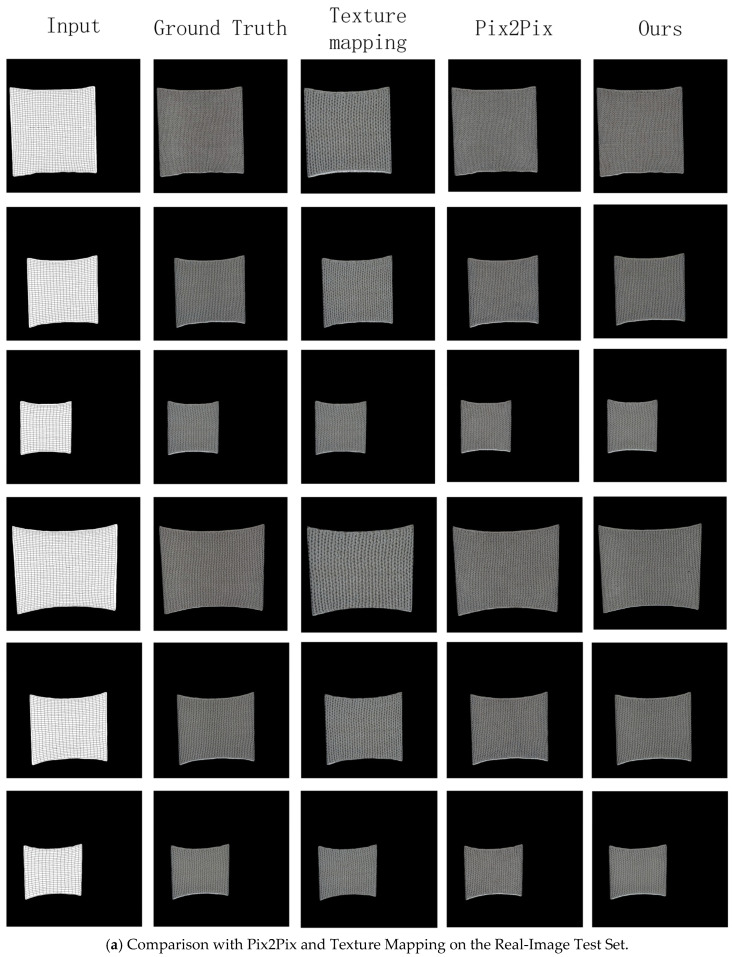

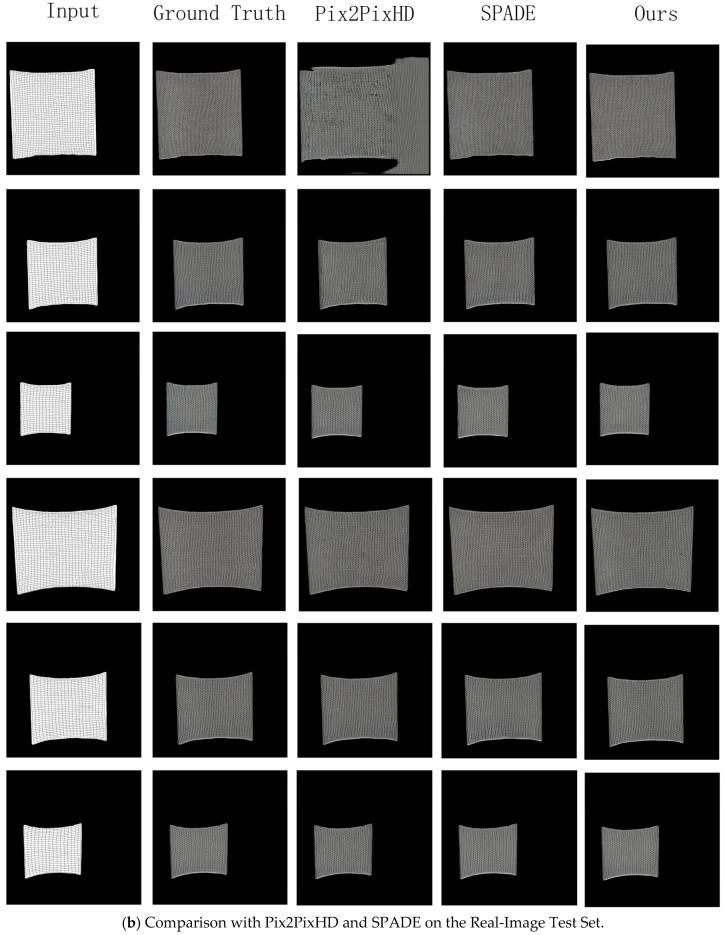

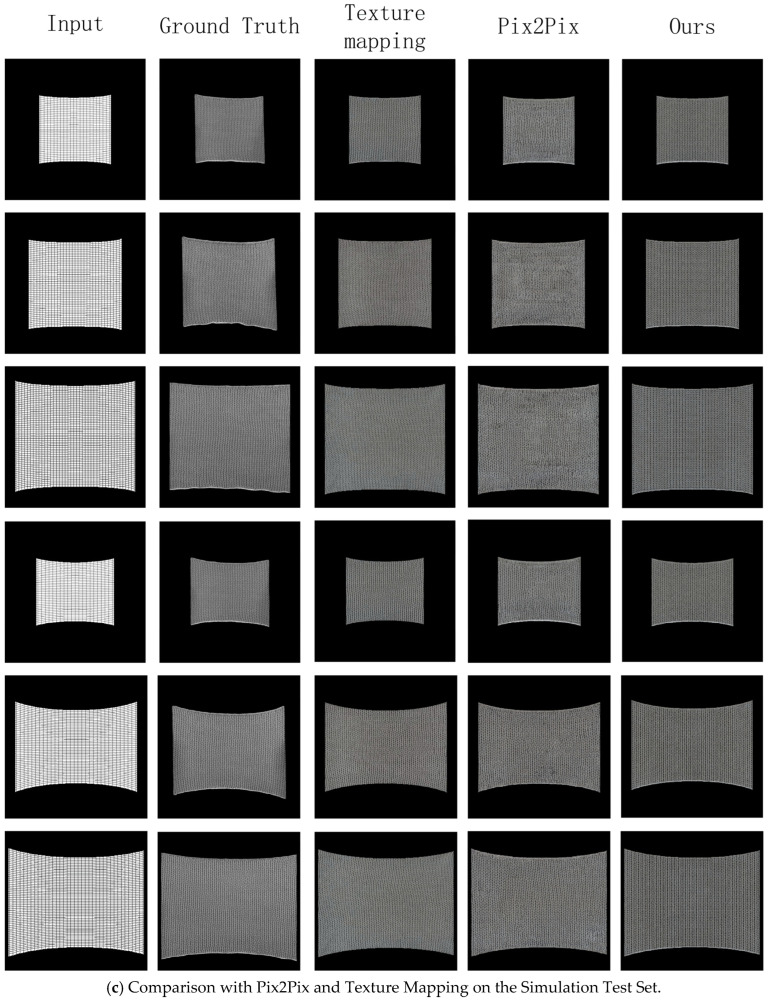

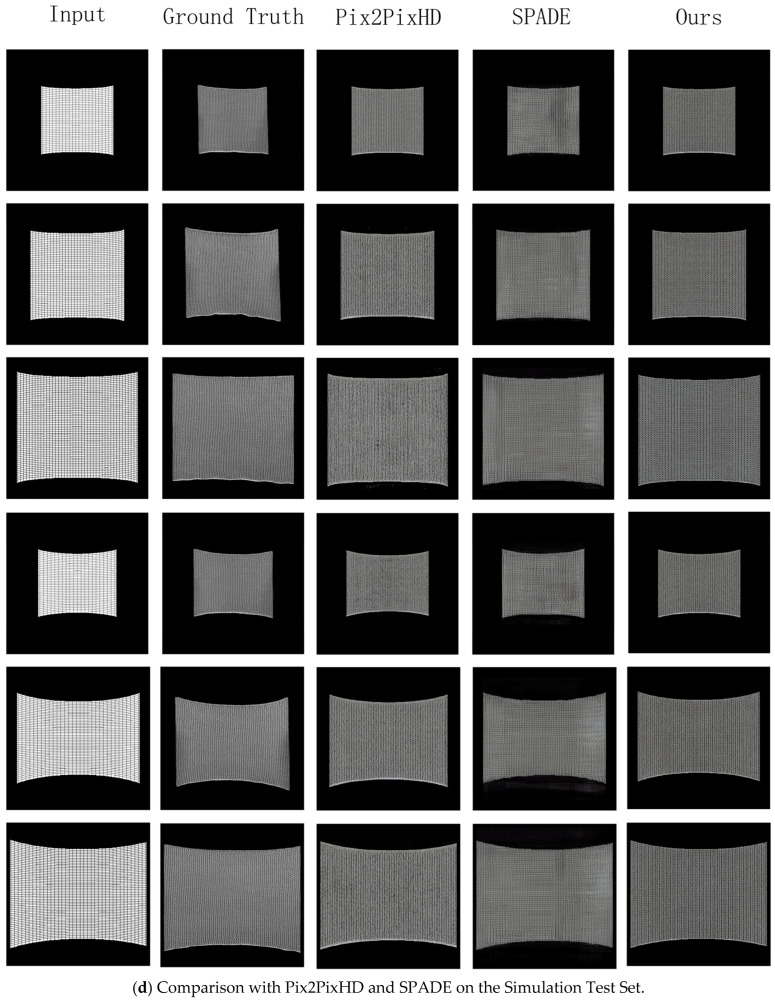
Comparison with various methods.

The visual comparisons are presented in Figure 6. Figure 6a,b show the results on the Real-Image Test Set, comparing our method with traditional texture mapping, the original Pix2Pix, Pix2PixHD, and SPADE models. Figure 6c,d present the corresponding comparisons on the Simulation Test Set, underlining the model’s performance when the input is a physically simulated mesh.

Qualitative analysis reveals the scenarios in which each method succeeds or fails. On the Real-Image Test Set, which contains deformation states similar to those seen during training, Pix2Pix, Pix2PixHD and SPADE are capable of producing plausible results in most cases. However, they frequently exhibit artifacts such as texture misalignment and discontinuities when tested on novel simulated deformation patterns. This issue is clearly visible in Figure 5. In contrast, our proposed method demonstrates consistent superiority across both test sets. It effectively solves the key issues of texture misalignment and inaccurate mapping, leading to significant improvements in visual quality, texture coherence, and, most importantly, generalization capability. This demonstrates that our framework is precisely tailored to address the fundamental objective of this work: generating plausible textures from arbitrary input mesh deformations.

To further objectively evaluate the performance of the methods, we use Peak Signal-to-Noise Ratio (PSNR), Structural Similarity Index (SSIM), and Learned Perceptual Image Patch Similarity (LPIPS) as quantitative evaluation metrics. we randomly sampled 5 subsets (each containing 10 input images) from the test set and computed the performance metrics for each model on these subsets independently. Table 3 and Table 4 details the comparison results of each method on the test set. On the simulation test set, the quantitative results further substantiate this advantage. Compared to traditional texture mapping methods, the PSNR value improves by 20.9%, SSIM by 21.8%, and LPIPS decreases by 24.3%. Compared to the original Pix2Pix model, the PSNR value improves by 26.1%, SSIM by 32.2%, and LPIPS decreases by 33.2%. These quantitative results demonstrate the effectiveness and superiority of the proposed method in the texture generation task for weft-knitted fabrics.

#### Perceptual User Study

While PSNR, SSIM, and LPIPS provide objective, pixel-level or feature-level comparisons, they cannot fully capture the perceptual realism of generated textures, which is the ultimate goal of our application. To address this, we conducted comprehensive, ranking-based user studies to obtain a direct, human-centric evaluation of visual quality on both our primary test sets: the Real-Image Test Set and the Spring-Mass Simulation Test Set. This dual-evaluation strategy allows us to assess the model’s performance on both fidelity to real data and generalization to simulated inputs. We designed a ranking task to gather granular preference data. (1) Real-Image Study: We selected 20 distinct knitted unit mesh maps from the Real-Image Test Set. For each, we prepared the Ground Truth (GT) real fabric image and textures generated by five methods. (2) Simulation Study: We selected 20 distinct knitted unit mesh maps from the Spring-Mass Simulation Test Set. Participants were shown the input knitted unit mesh maps at the top and asked to rank the 5 generated textures based on their plausibility as a realistic fabric deformation under the given mesh guidance. For both studies, each trial presented the reference (GT or knitted unit mesh maps) at the top, followed by 5 anonymized, randomized generated images. Participants ranked them from 1 (Best) to 5 (Worst). The studies were distributed via electronic questionnaire, collecting 200 valid responses per study. All images were displayed at 1024 × 1024 resolution.

The user study results, summarized in Table 5. On the Real-Image Test Set, learning-based models performed adequately, but the Texture Mapping method ranked lowest due to its obvious visual unnaturalness. The scenario reversed on the Simulation Test Set. Here, learning-based baselines often produced severe artifacts (misalignment, discontinuity), causing their rankings to drop. Texture Mapping, while unrealistic, avoided such glaring errors, placing it on par with or even above some learning models. In contrast, our Knit-Pix2Pix method consistently achieved the best Mean Rank on both sets, demonstrating superior visual plausibility and robust generalization.

**Figure 7:**
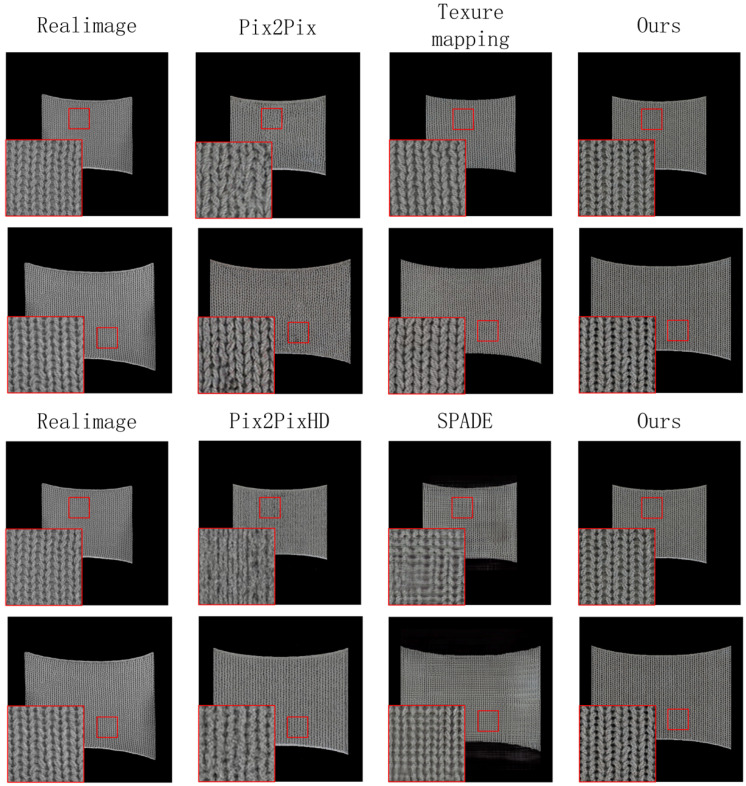
Detailed comparison. Red boxes indicate zoomed-in regions for local detail comparison.

Figure 7 further validates the technical advantages of our method at the microscopic detail level. In the localized magnification comparison of fabric textures, the Knit-Pix2Pix model demonstrates accurate generation of texture microstructures. Compared to traditional texture mapping techniques, the model can more accurately capture and reproduce the texture evolution patterns of fabrics during physical deformation, especially in the fabric’s stretched state, where it accurately reflects the morphological changes in the loop structure and its spatial relationships. Compared to the original Pix2Pix model, the improved model shows stronger texture coherence and realism, effectively addressing issues such as detail loss and texture misalignment. Overall analysis shows that the Knit-Pix2Pix network provides advantages in texture detail preservation, deformation consistency, and visual plausibility.

## 6. Discussion

The proposed Knit-Pix2Pix framework shows significant advantages over existing methods. Firstly, the Multi-Scale Feature Extraction (MSFE) module effectively addresses the limitations of fixed receptive fields in the traditional Pix2Pix network. By using paral-lel dilated convolutions with different dilation rates (1, 3, 7), the model can simultaneously capture local details, periodic patterns, and global deformation features, thus better un-derstanding the fabric’s multi-scale structural characteristics. The Grid-Guided Attention (GGA) mechanism uses structural information from the knitted unit mesh maps to guide feature learning. Unlike traditional attention mechanisms that rely solely on feature map statistics, GGA incorporates prior information about fabric deformation patterns. This de-sign excels in establishing the correspondence between fabric geometric changes and vis-ual texture changes. Furthermore, the multi-scale discriminator architecture further en-hances the quality of the generated textures by providing supervision at multiple resolu-tion levels. This design avoids common issues of detail loss and texture inconsistency in single-scale discriminators, especially when dealing with complex periodic structures such as knitted fabrics. Although the results are promising, there are areas in this study that can be further improved.

The method proposed in this study primarily addresses the texture generation task of weft-knitted fabrics under uniform bi-axial stretching conditions. The dataset was specifically designed for regular deformation patterns under controlled stretching environments, covering continuous deformation from the initial state to a 30% strain range. This method is suitable for applications requiring the simulation of fabric’s regular stretching deformation. For applications involving non-uniform deformation, local wrinkles, or twisting, further data expansion and model improvements are needed.

Overall, while this study has made significant progress in the realism of weft-knitted fabric texture generation, there is still room for improvement in areas such as generalization capability, providing clear directions for future research.

## 7. Conclusions

In this paper, we propose a Knit-Pix2Pix framework based on an improved Pix2Pix architecture, designed to generate fabric textures with high visual fidelity, structural consistency, and physical realism based on knitted unit mesh maps. By incorporating a Multi-Scale Feature Extraction (MSFE) module, Grid-Guided Attention (GGA) mechanism, and a multi-scale discriminator (MSD), the model significantly improves the realism of texture details. Experimental results show that, compared to traditional texture mapping methods, the proposed method improves PSNR by 20.9%, SSIM by 21.8%, and reduces LPIPS by 24.3%. Compared to the original Pix2Pix model, PSNR improves by 26.1%, SSIM increases by 32.2%, and LPIPS decreases by 33.2%. Ablation study results clearly demonstrate the effectiveness of each module in the Knit-Pix2Pix framework, validating the technical advantages of the proposed method. This study primarily validates the model’s performance under regular biaxial stretching, but it lacks further verification in more complex deformations. To significantly enhance the framework’s capabilities, future work will first involve testing its generalization on complex deformations (e.g., draping, collision) and different knitted structures or yarn materials. Furthermore, a central and promising future direction is the development of a multi-modal conditional generation framework, which aims to endow the system with greater versatility and precise control. We envision constructing an extended framework that can generate different weaving structures (e.g., plain knit, ribbed, reverse knit) selectively based on the same knitted unit mesh maps, using additional fabric type labels or feature vectors. This multi-modal input design will significantly enhance the model’s usability and adaptability, allowing a single model to cater to texture generation requirements for various fabric types. Furthermore, integrating material properties and lighting conditions into the generation process to enhance realistic rendering in virtual environments, along with incorporating temporal consistency for dynamic fabric simulation, are also important research directions worth exploring. These improvements will further drive the application development of fabric texture generation technology in fields such as computer graphics, virtual reality, and the textile industry.

## Figures and Tables

**Table 1:** Model complexity and efficiency comparison.

Method	Params	Training Time	Inference Time
Pix2Pix	**54.3 M**	**~26 h**	**~35 ms**
Pix2PixHD	97.5 M	~55 h	~68 ms
SPADE	92.1 M	~73 h	~80 ms
Ours (Knit-Pix2Pix)	70.2 M	~37 h	~46 ms

Note: Bold values indicate the best performance under the corresponding condition.

**Table 2:** Ablation experimental results (Dev. over 5 random Simulation Test Set subsets).

ID	Base	MSFE	GGA	MSD	PSNR (↑)	SSIM (↑)	LPIPS (↓)
M1	✓	✗	✗	✗	16.853 ± 2.436	0.601 ± 0.117	0.201 ± 0.024
M2	✓	✗	✓	✓	17.991 ± 2.501	0.630 ± 0.145	0.183 ± 0.020
M3	✓	✓	✗	✓	18.605 ± 2.319	0.703 ± 0.188	0.167 ± 0.018
M4	✓	✓	✓	✗	20.155 ± 2.905	0.750 ± 0.175	0.149 ± 0.015
M5 (Ours)	✓	✓	✓	✓	**20.821** ± **2.892**	**0.787** ± **0.196**	**0.143** ± **0.029**

Note: Bold values indicate the best performance under the corresponding condition.

**Table 3:** Quantitative results (Dev. over 5 random Real-Image Test Set subsets).

Method	PSNR (↑)	SSIM (↑)	LPIPS (↓)
Texture Mapping	17.974 ± 2.643	0.695 ± 0.133	0.121 ± 0.040
Pix2Pix	22.564 ± 5.778	0.770 ± 0.117	0.092 ± 0.018
Pix2PixHD	22.616 ± 3.756	0.790 ± 0.229	0.091 ± 0.019
SPADE	24.721 ± 2.655	0.852 ± 0.066	0.085 ± 0.015
Ours (Knit-Pix2Pix)	**25.821** ± **2.892**	**0.877** ± **0.059**	**0.083** ± **0.014**

Note: Bold values indicate the best performance under the corresponding condition.

**Table 4:** Quantitative results (Dev. over 5 random Simulation Test Set subsets).

Method	PSNR (↑)	SSIM (↑)	LPIPS (↓)
Texture Mapping	17.025 ± 2.502	0.643 ± 0.165	0.184 ± 0.025
Pix2Pix	16.853 ± 2.436	0.601 ± 0.117	0.201 ± 0.024
Pix2PixHD	18.233 ± 1.969	0.638 ± 0.164	0.198 ± 0.033
SPADE	18.114 ± 2.495	0.648 ± 0.166	0.185 ± 0.030
Ours (Knit-Pix2Pix)	**20.821** ± **2.892**	**0.787** ± **0.196**	**0.143** ± **0.029**

Note: Bold values indicate the best performance under the corresponding condition.

**Table 5:** Perceptual user study.

Method	Mean Rank (↓) (Real-Image Test Set.)	Mean Rank (↓) (Simulation Test Set)
Texture Mapping	3.85 ± 0.50	3.45 ± 0.50
Pix2Pix	3.25 ± 0.45	3.50 ± 0.48
Pix2PixHD	3.05 ± 0.40	3.65 ± 0.45
SPADE	3.20 ± 0.42	3.70 ± 0.47
Ours (Knit-Pix2Pix)	**2.20** ± **0.35**	**1.95** ± **0.41**

Note: Bold values indicate the best performance under the corresponding condition.

## Data Availability

The data and code that support the findings of this study are not publicly available due to confidentiality and proprietary restrictions. However, they may be available from the corresponding author upon reasonable request and with permission from the funding institutions.
